# Supplementary material for: IFT proteins interact with HSET to promote supernumerary centrosome clustering in mitosis
Source: EMBO Rep. 2020 Apr 9;21(6):e49234. doi: 10.15252/embr.201949234 (PMC7271317; doi:10.15252/embr.201949234)
Supplement: Supplementary file 1 — Expanded View Figures PDF [file EMBR-21-e49234-s001.pdf]

Expanded View Figures

Figure EV1. IFT proteins contribute to centrosome clustering in RPE-1 cells.

A Quantification of centrosome amplification in the tetracycline/doxycycline-inducible cell lines used in the different experiments, with or without doxycycline treatment. Quantification of centrosome amplification in diploid (2N) and tetraploid (4N) HCT-116 cell lines is also shown. A centrosome corresponds to two centrin dots colocalizing with gamma-tubulin signal. Bars represent the mean of a minimum of three independent experiments ± SEM. For each cell line and condition, a minimum of 257 cells were quantified.

B Immunofluorescence images of mitotic RPE-1 cells, with supernumerary centrosomes, highlighting IFT88 localization in mitosis. Magnified boxes, spindle poles. Scale bar in magnified box, 1 μm.

C Immunoblots showing IFT proteins after IFT52 depletion in RPE-1 cells. IFT52 depletion was rescued by re-expressing mouse mcherry IFT52 in the cells. GAPDH, loading control.

D Quantification of the percentage of multipolar anaphases following the indicated siRNA treatments in RPE-1 cells expressing H2B-GFP with centrosome amplification. Mean ± SEM from three independent experiments. $*P < 0.05$; unpaired t-tests. For each condition, a minimum of 499 cells were quantified.

E Still images from a movie of RPE-1 cells with centrosome amplification that stably express H2B-GFP. Control and siRNA depletion are shown.

F Immunoblots showing IFT proteins after the indicated siRNA treatments in RPE-1 cells with centrosome amplification. GAPDH, loading control.

G Quantification of the percentage of multipolar anaphases following the indicated siRNA treatment in RPE-1 cells with centrosome amplification. Mean ± SEM from four independent experiments. $*P < 0.05$; $**P < 0.01$; ns: not significant. unpaired t-test. For each condition, a minimum of 909 cells were quantified.

H Immunofluorescence images of mitotic RPE-1 cells, with or without supernumerary centrosomes and with or without IFT88 siRNA treatment. Magnified boxes, spindle poles. Scale bar in magnified box, 1 μm.

I Quantification of the percentage of multipolar mitosis in RPE-1 cells without centrosome amplification and treated with the indicated siRNA. Mean ± SEM from three independent experiments. ns: not significant; unpaired t-test. For each condition, a minimum of 245 cells were quantified.

J Quantification of the percentage of spindle poles presenting less than two centrioles in RPE-1 cells with centrosome amplification and treated with the indicated siRNA. Mean ± SEM from three independent experiments. ns: not significant; unpaired t-test. For each condition, a minimum of 155 cells were quantified.

K Immunofluorescence images of mitotic RPE-1 cells, with supernumerary centrosomes and with or without IFT88 siRNA treatment. Centrosomes are double-labeled with centrin-EGFP and polyglutamylated tubulin, using GT335 antibody. Magnified boxes, spindle poles. Scale bar in magnified box, 1 μm.

Source data are available online for this figure.

© 2020 The Authors

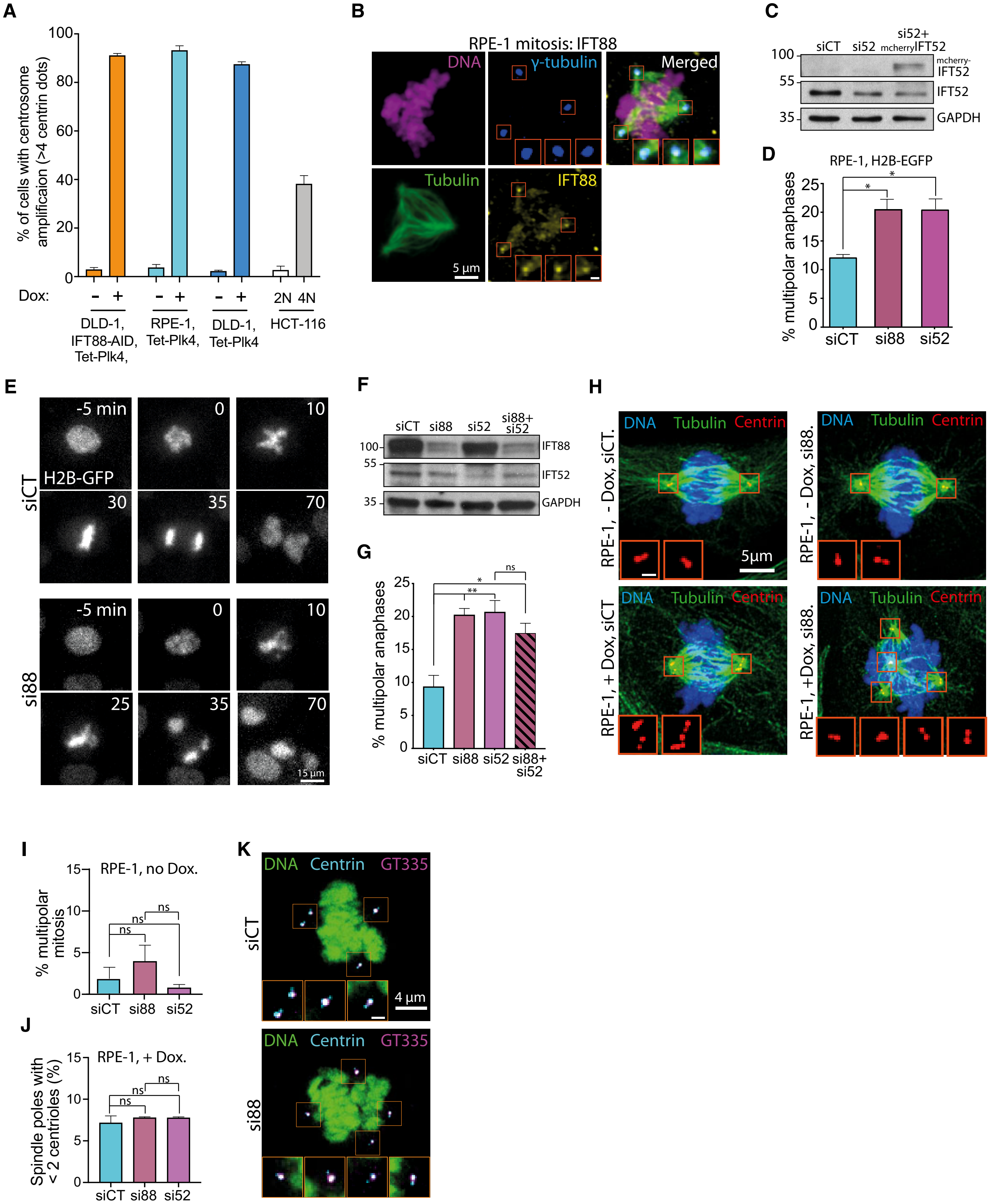

A
% of cells with centrosome amplificaion (>4 centrin dots)
Dox:
DLD-1, IFT88-AID, Tet-Plk4,
RPE-1, Tet-Plk4,
DLD-1, Tet-Plk4
HCT-116
2N 4N
B
RPE-1 mitosis: IFT88
DNA
γ-tubulin
Merged
Tubulin
IFT88
5 µm
C
siCT
si52
si52+ mcherryIFT52
mcherry-IFT52
IFT52
GAPDH
D
RPE-1, H2B-EGFP
% multipolar anaphases
siCT
si88
si52
E
H2B-GFP
siCT
si88
-5 min
15 µm
F
siCT si88 si52 si88+ si52
IFT88
IFT52
GAPDH
G
% multipolar anaphases
ns
H
DNA Tubulin Centrin
RPE-1, - Dox, siCT.
RPE-1, - Dox, si88.
RPE-1, + Dox, siCT
RPE-1, +Dox, si88.
5µm
I
RPE-1, no Dox.
% multipolar mitosis
J
RPE-1, + Dox.
Spindle poles with < 2 centrioles (%)
K
DNA Centrin GT335
siCT
si88
4 µm

Figure EV1.

Figure EV2. IFT proteins contribute to centrosome clustering in association with the mitotic motor HSET.

A Immunofluorescence images of mitotic DLD-1 cells targeted with an AID tag on IFT88, with or without supernumerary centrosomes, and with or without auxin treatment. Magnified boxes, spindle poles. Scale bar in magnified box, 1 μm.

B Immunofluorescence images of mitotic DLD-1, with or without supernumerary centrosomes and treated with the indicated siRNA. Magnified boxes, spindle poles. Scale bar in magnified box, 1 μm.

C Top, immunoblots showing IFT88 depletion in DLD-1 cells following the indicated siRNA treatment. Bottom, quantification of unclustered centrosomes in mitotic DLD-1 cells with supernumerary centrosomes following 30 h of siRNA treatment. Mean ± SEM from a minimum of three independent experiments. $^{**}P < 0.01$; unpaired *t*-test. For each condition, a minimum of 192 cells were quantified.

D Immunofluorescence images of mitotic HCT-116 diploid (2N) and tetraploid cells (4N), treated with the indicated siRNA. Magnified boxes, spindle poles. Scale bar in magnified box, 1 μm.

E Top, immunoblots showing IFT88 depletion in HCT-116 cells following the indicated siRNA treatment. Bottom, quantification of the percentage of unclustered centrosomes in mitotic diploid and tetraploid HCT-116 following 30 h of siRNA treatment. Mean ± SEM from a minimum of three independent experiments. $^{*}P < 0.01$; unpaired *t*-test. For each condition, a minimum of 156 cells were quantified.

F Quantification of the percentage of spindle poles presenting less than two centrioles in DLD-1 cells with centrosome amplification and treated with or without auxin. Mean ± SEM from three independent experiments. ns: not significant; unpaired *t*-test. For each condition, a minimum of 152 cells were quantified.

G Quantification of the percentage of multipolar mitosis in DLD-1 cells without centrosome amplification and treated with or without auxin. Mean ± SEM from three independent experiments. ns: not significant; unpaired *t*-test. For each condition, a minimum of 164 cells were quantified.

H Immunoblots showing IFT proteins after the indicated siRNA treatments in RPE-1 cells. GAPDH, loading control.

I Quantification of the percentage of multipolar anaphases following the indicated siRNA treatments. Mean ± SEM from three independent experiments. $^{****}P < 0.0001$; $^{**}P < 0.01$; ns: non-significant; unpaired *t*-test. For each condition, a minimum of 542 cells were quantified.

Source data are available online for this figure.

© 2020 The Authors

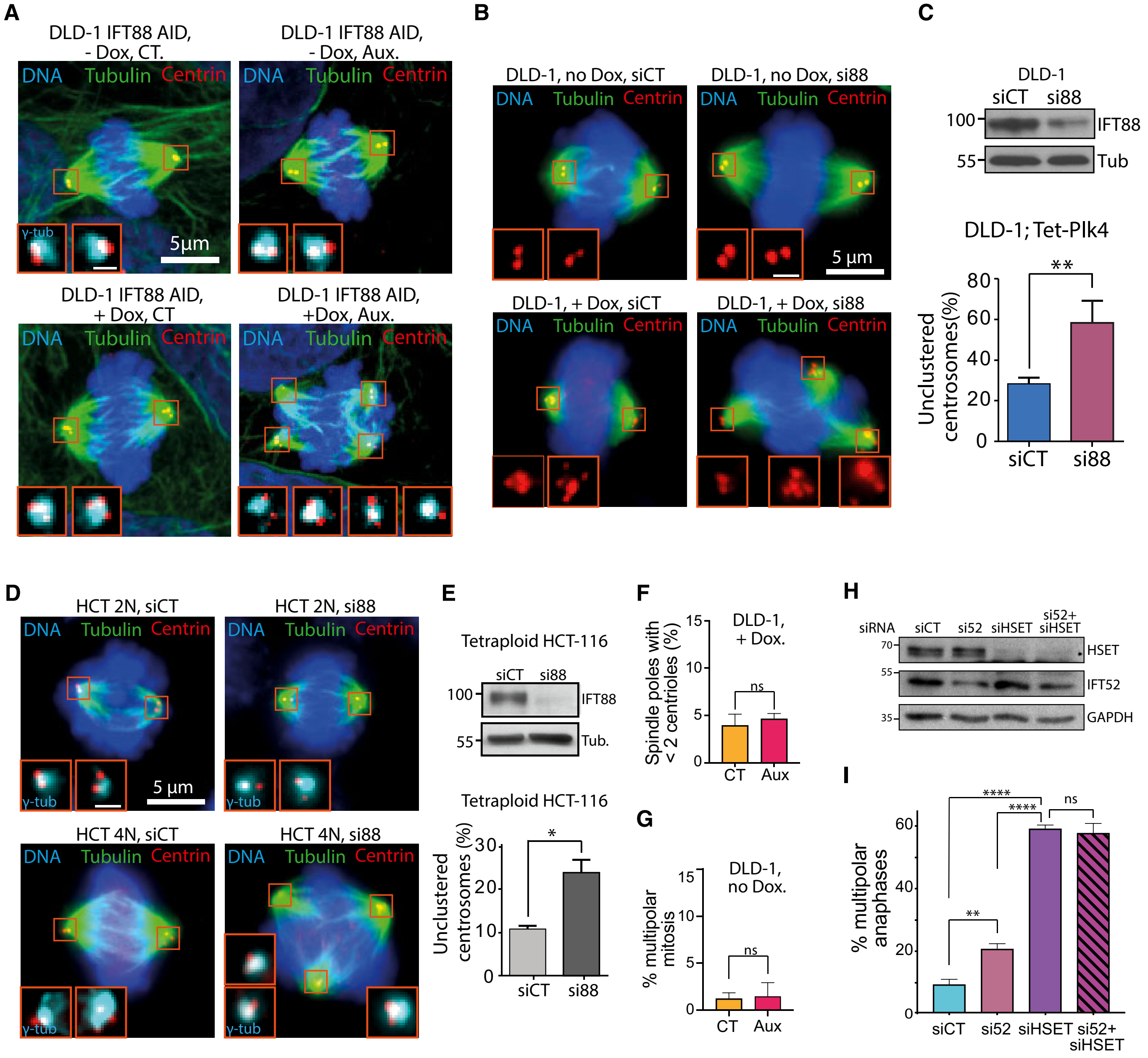
A
DLD-1 IFT88 AID, - Dox, CT.
DLD-1 IFT88 AID, - Dox, Aux.
DLD-1 IFT88 AID, + Dox, CT
DLD-1 IFT88 AID, +Dox, Aux.
DNA Tubulin Centrin
γ-tub
5µm
B
DLD-1, no Dox, siCT
DLD-1, no Dox, si88
DLD-1, + Dox, siCT
DLD-1, + Dox, si88
5 µm
C
DLD-1
siCT si88
100
55
IFT88
Tub
DLD-1; Tet-Plk4
Unclustered centrosomes(%)
**
siCT si88
D
HCT 2N, siCT
HCT 2N, si88
HCT 4N, siCT
HCT 4N, si88
5 µm
E
Tetraploid HCT-116
siCT si88
IFT88
Tub.
Unclustered centrosomes (%)
*
F
Spindle poles with < 2 centrioles (%)
DLD-1, + Dox.
ns
CT Aux
G
% multipolar mitosis
DLD-1, no Dox.
ns
CT Aux
H
siRNA siCT si52 siHSET si52+ siHSET
HSET
IFT52
GAPDH
I
% multipolar anaphases
****
****
ns
**
siCT si52 siHSET si52+ siHSET

Figure EV2.

Figure EV3. Cancer cells naturally harboring supernumerary centrosomes depend on IFT proteins for proper proliferation.

A Quantification of the fluorescence signal of the GFP-HSET in DLD-1 cells, at the last time point before photobleaching for the indicated conditions. The horizontal line is the mean intensity of four independent experiments. ns: not significant. Unpaired *t*-test. A minimum of 41 measurements, per condition, were tested.
B Still images from live imaging of RPE-1 cells with centrosome amplification (+dox) expressing centrin-EGFP. The two images displayed were acquired at 1 min interval with a 40× and a 100× objectives (left and right, respectively). The DNA is labeled with SiR-DNA. Magnified boxes, centrioles. Scale bar in magnified box, 1 μm.
C Left panel, temporal color-coded tracks of EB1 comets in a RPE-1 cell stably expressing EB1-GFP and its corresponding computed tracks (central panel) using TrackMate plugin (ImageJ). Right, quantification of the average velocity of EB1 comets in RPE-1 cells stably expressing EB1-GFP treated with the indicated conditions. Mean ± SEM of two independent experiments. A minimum of 1,014 comets were analyzed for each condition.
D Immunoblots showing IFT proteins after the indicated siRNA treatments in Caco-2 cells. IFT52 depletion was rescued by re-expressing mouse mcherry IFT52 in the cells. GAPDH, loading control.
E Immunofluorescence images of mitotic figures in Caco-2 and MDA-MB-231 cells with normal and abnormal centrosome numbers. Magnified boxes, spindle poles. Scale bar in magnified box, 1 μm.
F Quantification of the clonogenic capability of DLD-1 cells harboring, or not, supernumerary centrosomes upon doxycycline induction of Plk4 and treated with the indicated siRNA. siCT is normalized to 100%. Mean ± SEM of two independent experiments. For each experiment, each condition was done in duplicate.
G Representative image of clones of DLD-1 cells, treated with the indicated conditions and stained with crystal violet.
H Quantification of the clonogenic capability of diploid and tetraploid HCT-116 cells treated with the indicated siRNA. siCT is normalized to 100%. Mean ± SEM of two independent experiments done in duplicate.
I Representative image of clones of diploid and tetraploid HCT-116 cells, treated with the indicated conditions and stained with crystal violet.

Source data are available online for this figure.

 © 2020 The Authors

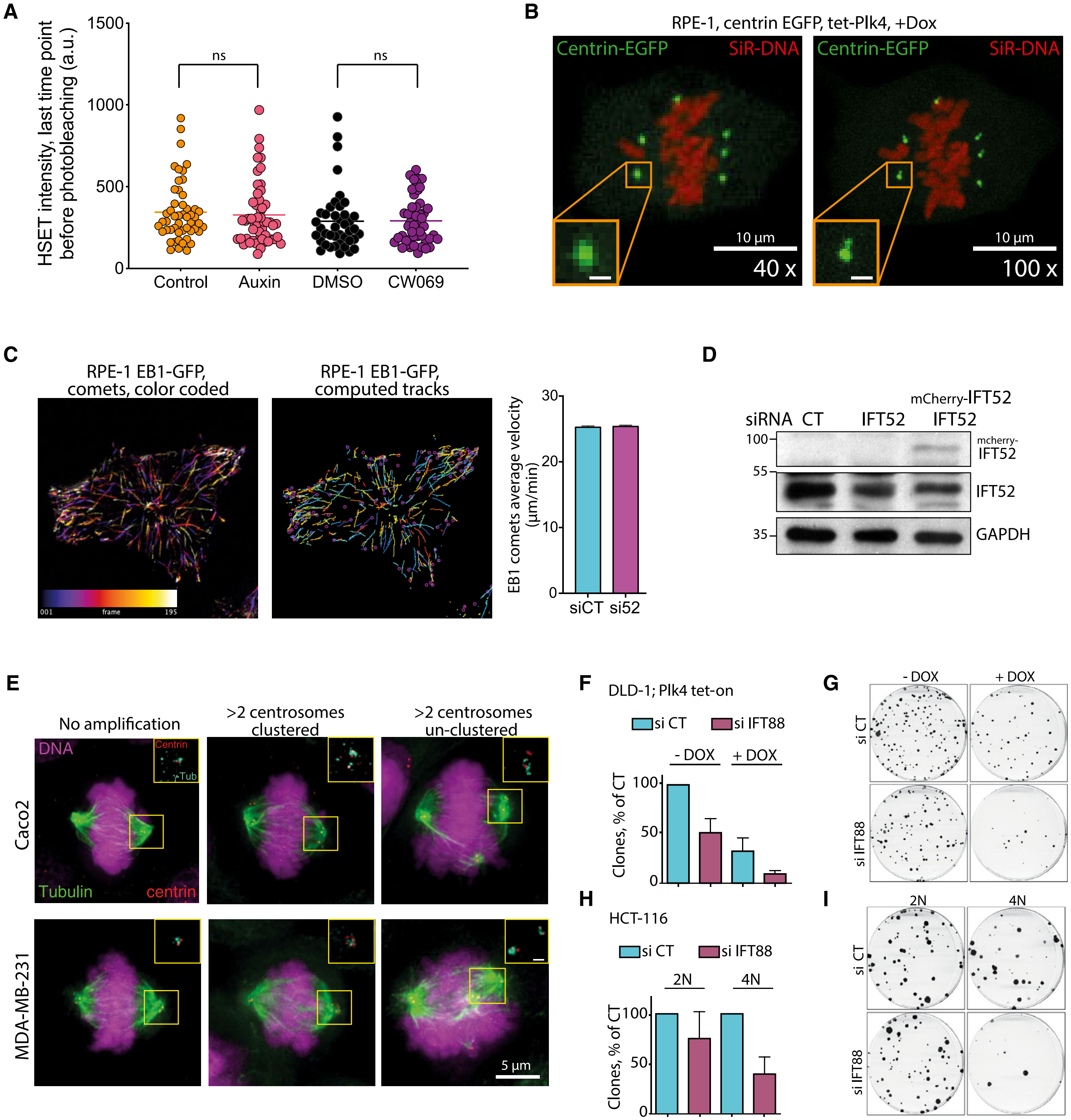

A
HSET intensity, last time point before photobleaching (a.u.)
1500
1000
500
0
ns
ns
Control
Auxin
DMSO
CW069
B
RPE-1, centrin EGFP, tet-Plk4, +Dox
Centrin-EGFP
SiR-DNA
10 µm
40 x
100 x
C
RPE-1 EB1-GFP, comets, color coded
RPE-1 EB1-GFP, computed tracks
EB1 comets average velocity (µm/min)
30
20
10
0
siCT
si52
D
mCherry-IFT52
siRNA CT IFT52 IFT52
100
55
35
mcherry-IFT52
IFT52
GAPDH
E
No amplification
>2 centrosomes clustered
>2 centrosomes un-clustered
DNA
Centrin
γ-Tub
Caco2
Tubulin
centrin
MDA-MB-231
5 µm
F
DLD-1; Plk4 tet-on
si CT
si IFT88
- DOX
+ DOX
Clones, % of CT
100
50
0
G
- DOX
+ DOX
si CT
si IFT88
H
HCT-116
si CT
si IFT88
2N
4N
Clones, % of CT
100
50
0
I
2N
4N
si CT
si IFT88

Figure EV3.

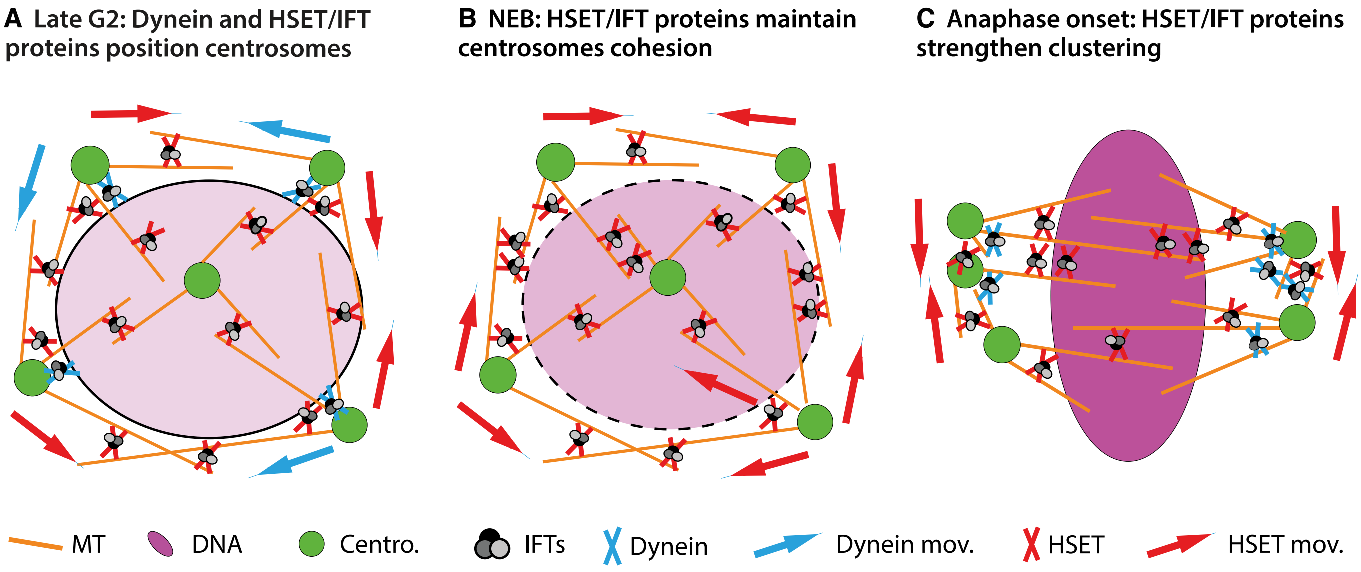

Figure EV4. Molecular model of IFT proteins functions together with HSET for centrosome clustering during mitosis progression.

A In late G2, dynein helps to maintain the contact between supernumerary centrosomes and the nuclear envelope. Both HSET and dynein, together with IFT proteins, mediate supernumerary centrosome movements around the nucleus.

B At NEB, dynein/nuclear envelope contacts are lost, HSET together with IFT proteins contribute to maintain inter-centrosomal cohesion. In the absence of IFT proteins, this activity of HSET is reduced and the average distance between adjacent centrosome increases (gray arrowhead Fig 4D).

C During mitosis, both dynein and HSET, together with IFT proteins, contribute to focus supernumerary centrosomes into spindle poles. In the absence of IFT proteins, this activity is reduced, at least for HSET, resulting in an increased inter-centrosomal distance (black arrowhead Fig 4D). Eventually, spindles fail to organize into a bipolar structure, and, in anaphase, the DNA is segregated in more than two DNA mass due to multipolar spindle organization (Figs 1 and EV1 and 4). This abnormal DNA segregation results in reduced cell proliferation in cells naturally harboring supernumerary centrosomes (Fig 5). MT: microtubules. Centro.: centrosomes. Dynein mov.: centrosome movements resulting from dynein activity. HSET mov.: centrosome movements resulting from HSET activity.

 © 2020 The Authors
