# Supplementary material for: IFT proteins interact with HSET to promote supernumerary centrosome clustering in mitosis
Source: EMBO Rep. 2020 Apr 9;21(6):e49234. doi: 10.15252/embr.201949234 (PMC7271317; doi:10.15252/embr.201949234)
Supplement: Supplementary file 2 — Movie EV1 [file EMBR-21-e49234-s002.zip › Movie EV1/Movie EV1.pdf]

Movie EV1

Live imaging of a bipolar anaphase in RPE-1 cell treated with siCT. See Fig 1 for stills and description. Display rate, 5 frames/ sec.
