# Supplementary material for: IFT proteins interact with HSET to promote supernumerary centrosome clustering in mitosis
Source: EMBO Rep. 2020 Apr 9;21(6):e49234. doi: 10.15252/embr.201949234 (PMC7271317; doi:10.15252/embr.201949234)
Supplement: Supplementary file 4 — Movie EV3 [file EMBR-21-e49234-s004.zip › Movie EV3/Movie EV3.pdf]

Movie EV3

Live imaging of a bipolar anaphase in DLD-1 cell in control condition. See Fig 1 for stills and description. Display rate, 5 frames/ sec.
