# Supplementary material for: IFT proteins interact with HSET to promote supernumerary centrosome clustering in mitosis
Source: EMBO Rep. 2020 Apr 9;21(6):e49234. doi: 10.15252/embr.201949234 (PMC7271317; doi:10.15252/embr.201949234)
Supplement: Supplementary file 5 — Movie EV4 [file EMBR-21-e49234-s005.zip › Movie EV4/Movie EV4.pdf]

Movie EV4

Live imaging of a multipolar anaphase in DLD-1 cell treated with auxin. See Fig 1 for stills and description. Display rate, 5 frames/ sec.
