# Supplementary material for: IFT proteins interact with HSET to promote supernumerary centrosome clustering in mitosis
Source: EMBO Rep. 2020 Apr 9;21(6):e49234. doi: 10.15252/embr.201949234 (PMC7271317; doi:10.15252/embr.201949234)
Supplement: Supplementary file 8 — Movie EV7 [file EMBR-21-e49234-s008.zip › Movie EV7/Movie EV7.docx]

**Movie EV7**

Live imaging of a multipolar anaphase and centrosome behavior in RPE-1 cell in si52 condition. See Fig 4 for stills and description. Display rate, 10 frames/ sec.
