# Supplementary figures and images for: IFT proteins interact with HSET to promote supernumerary centrosome clustering in mitosis
Source: EMBO Rep. 2020 Apr 9;21(6):e49234. doi: 10.15252/embr.201949234 (PMC7271317; doi:10.15252/embr.201949234)

Figure EV2C

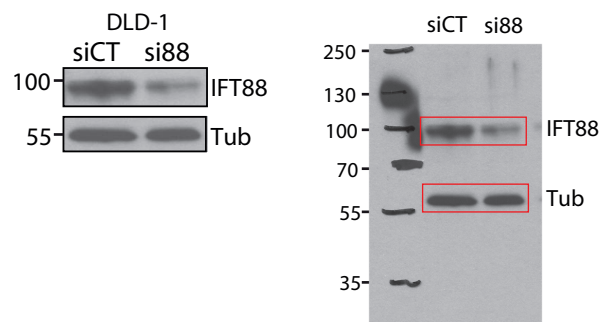

Figure EV2E

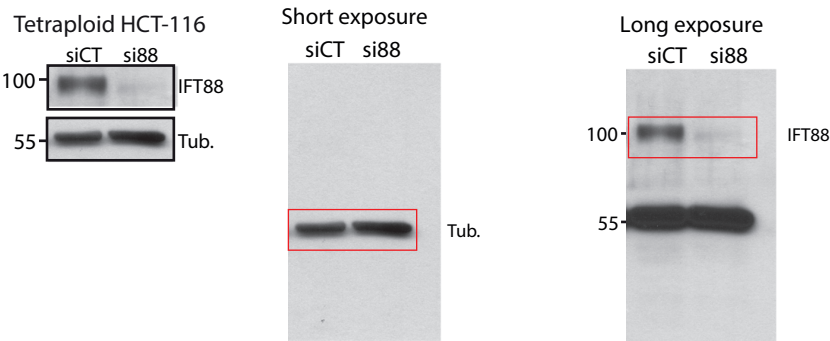

Figure EV2H

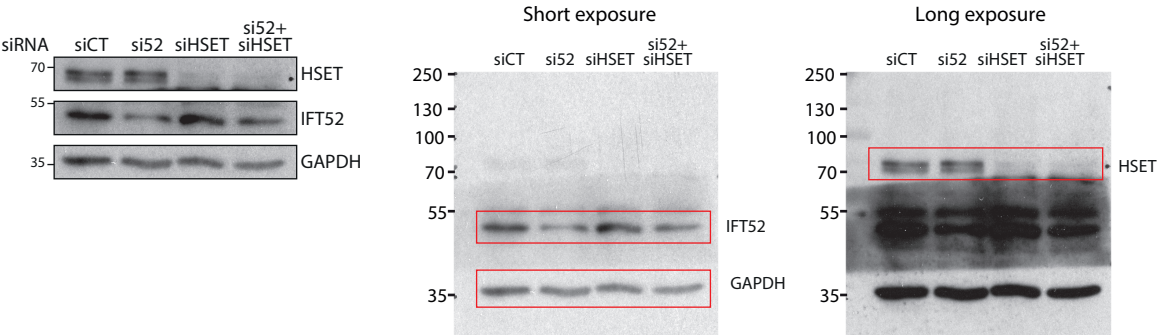

Supplement: Supplementary file 9 — Source Data for Expanded View [file EMBR-21-e49234-s013.zip › Source_data_Figure_EV2_manuscript_EMBOR-2019-49234V3.pdf]

Figure EV3D

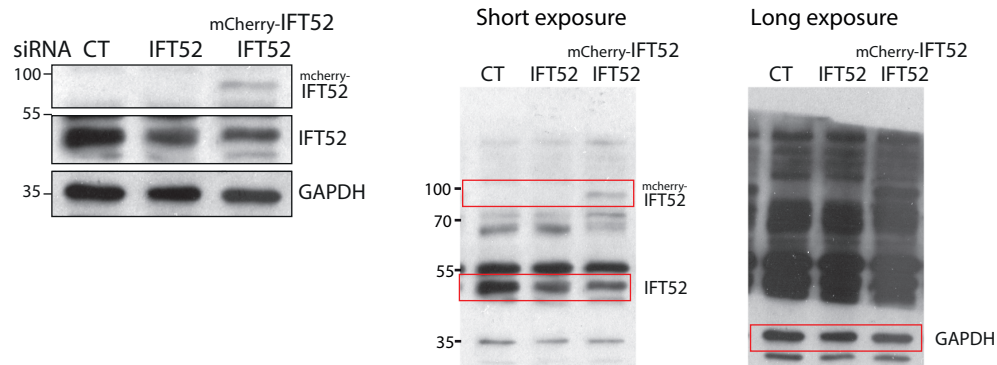

Supplement: Supplementary file 9 — Source Data for Expanded View [file EMBR-21-e49234-s013.zip › Source_data_Figure_EV3_manuscript_EMBOR-2019-49234V3.pdf]

Figure EV1C

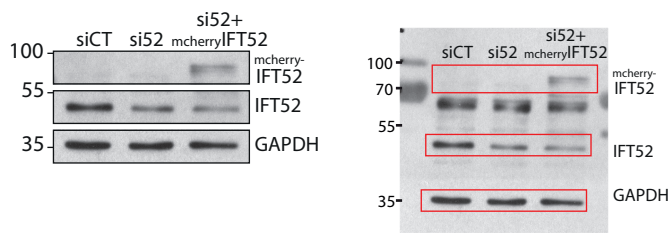

Figure EV1F

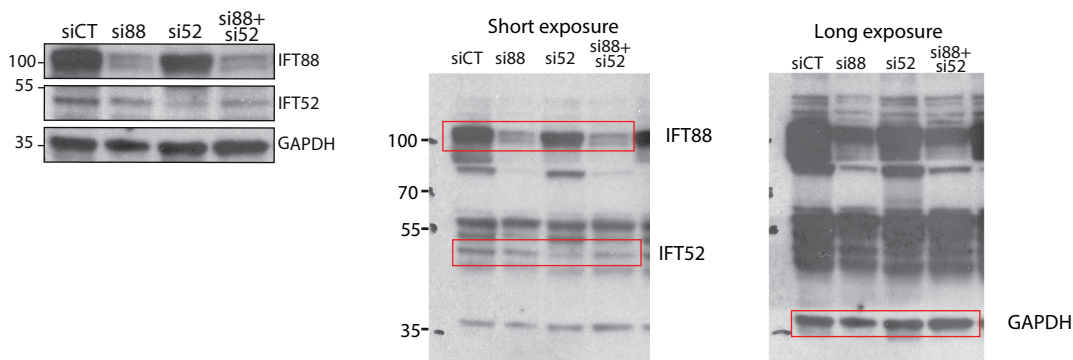

Supplement: Supplementary file 9 — Source Data for Expanded View [file EMBR-21-e49234-s013.zip › Source_data_Figure_EV1_manuscript_EMBOR-2019-49234V3.pdf]

Source data Figure 1

Figure 1D

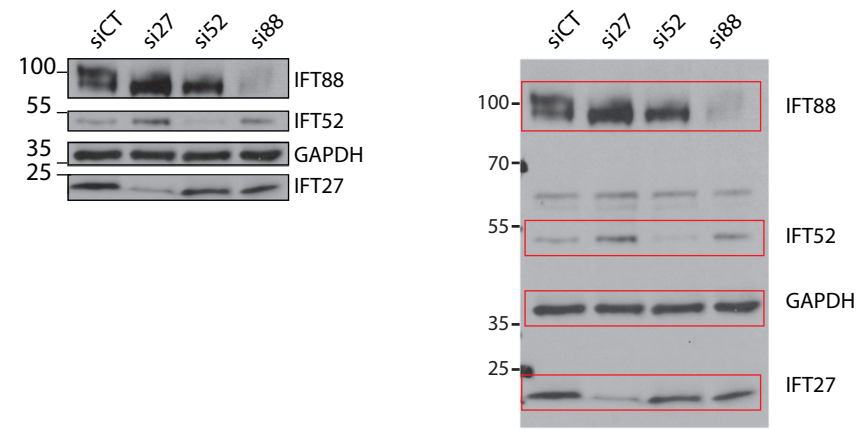

Figure 1G

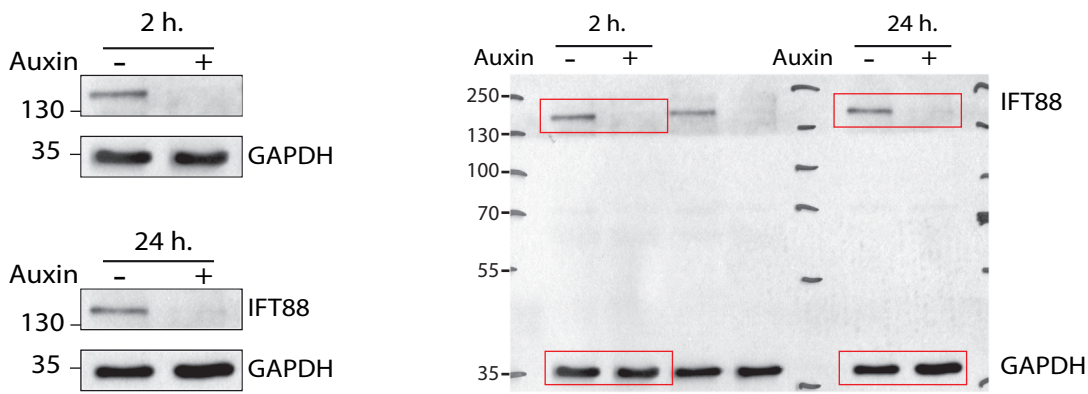

Supplement: Supplementary file 11 — Source Data for Figure 1 [file EMBR-21-e49234-s009.pdf]

Figure 2E

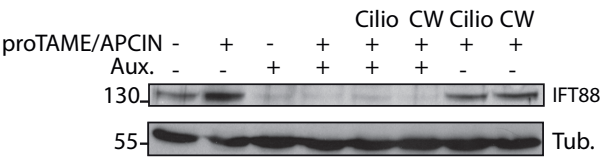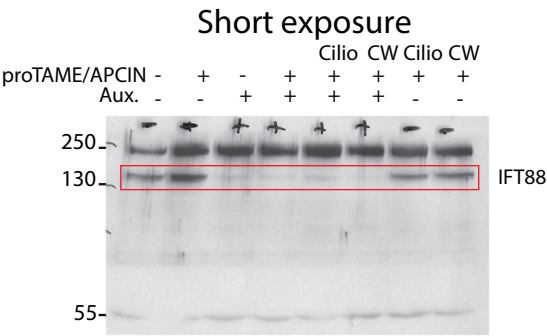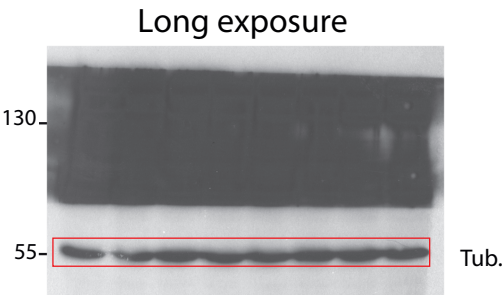

Supplement: Supplementary file 12 — Source Data for Figure 2 [file EMBR-21-e49234-s010.pdf]

## Source data Figure 3

Figure 3B

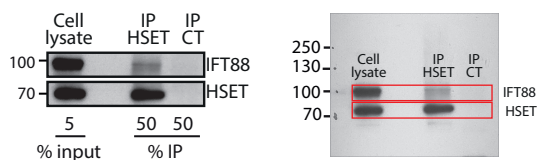

Figure 3D

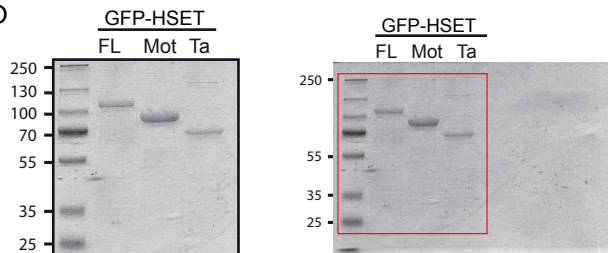

Figure 3E

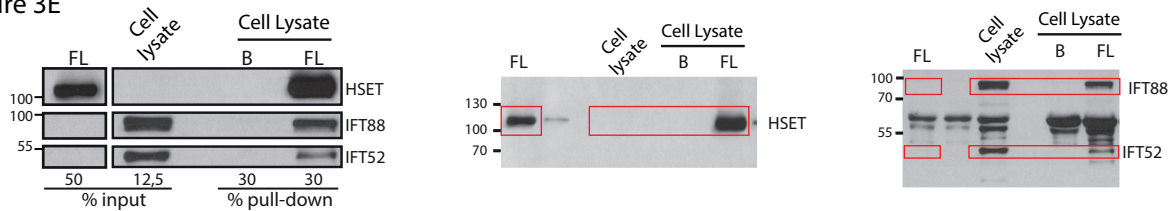

Figure 3F

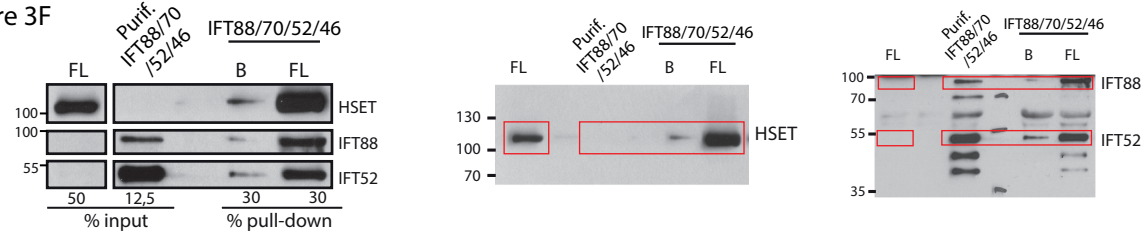

Figure 3G

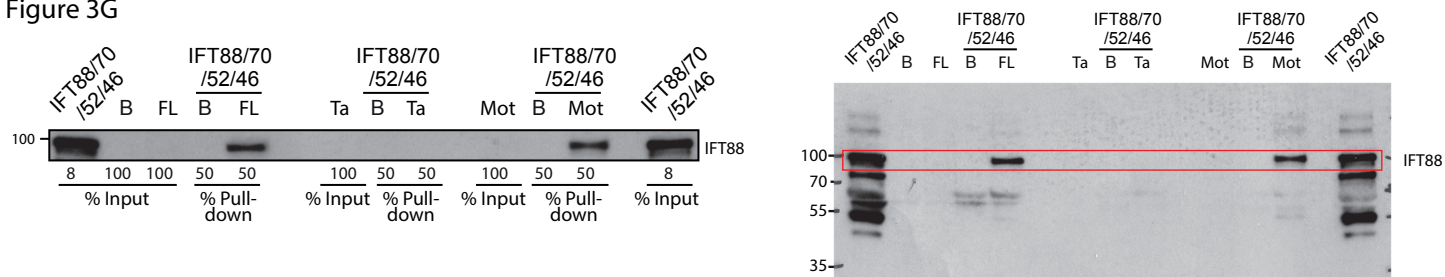

Figure 3H

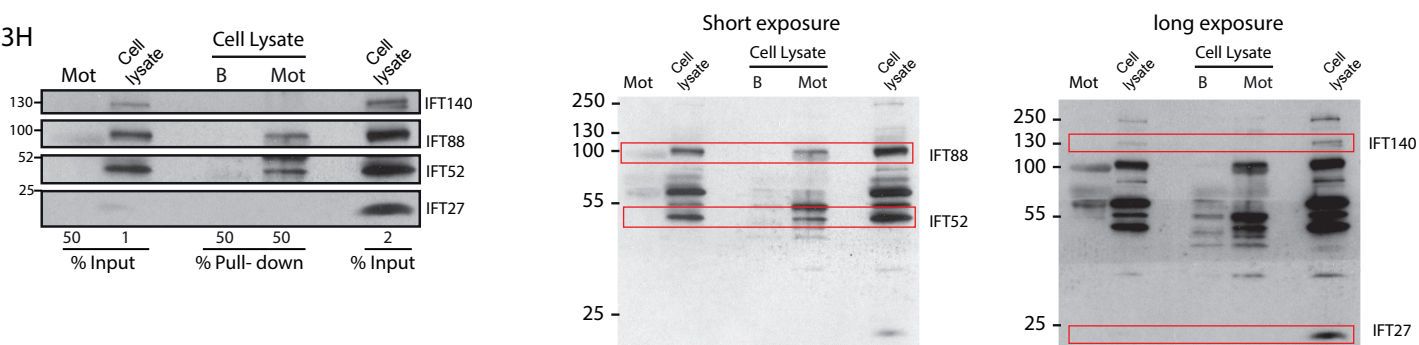

Supplement: Supplementary file 13 — Source Data for Figure 3 [file EMBR-21-e49234-s011.pdf]

Source data Figure 5

Source data Figure 5C

Figure 5C

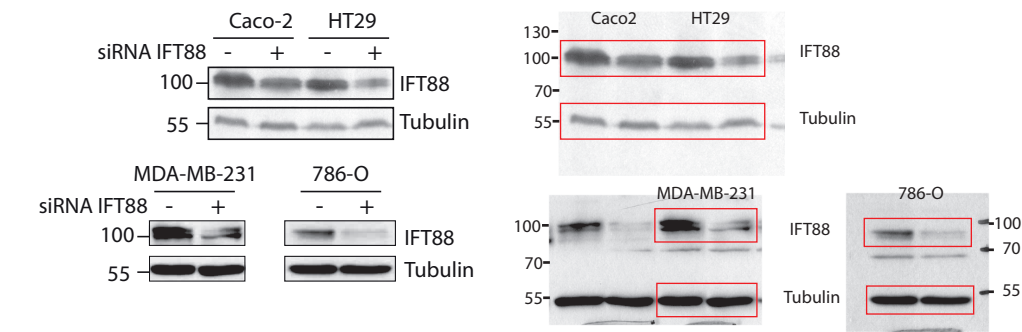

Figure 5I

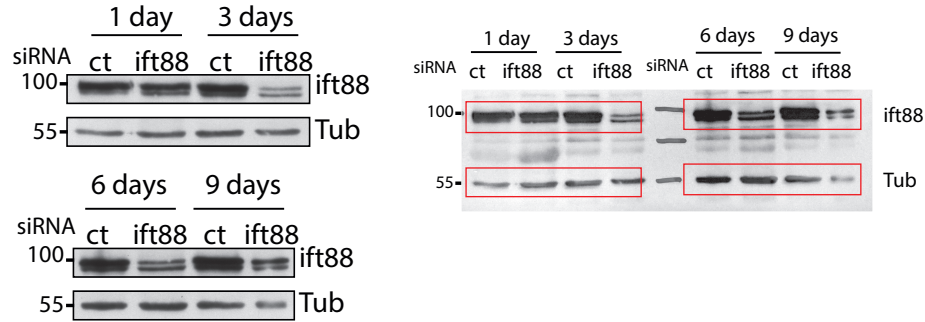

Supplement: Supplementary file 14 — Source Data for Figure 5 [file EMBR-21-e49234-s012.pdf]
